# A Horizon Scan of Neurotechnology Innovations

**DOI:** 10.3390/ijerph22050811

**Published:** 2025-05-21

**Authors:** Shona Haston, Sean Gill, Katie Twentyman, Elizabeth Green, Opeyemi Agbeleye, Claire Eastaugh, Dawn Craig, Sonia Garcia Gonzalez-Moral, Andrew Mkwashi

**Affiliations:** National Institute for Health and Care Research (NIHR) Innovation Observatory, Population Health Sciences Institute, Faculty of Medical Sciences, Newcastle University, Newcastle upon Tyne NE1 7RU, UK

**Keywords:** neurotechnology, horizon scanning, mental health, healthy ageing, physical disability

## Abstract

Neurotechnology is a rapidly emerging field with vast potential within healthcare, but also has inherent concerns. There is, therefore, a need to ensure the responsible and ethical development and regulation of these technologies. This horizon scan aimed to provide an overview of neurotechnologies in development and those approved by the FDA as of June 2024 for a range of conditions relating to mental health, healthy ageing, and physical disability. Searches of clinical trials, conferences, journals, and news were performed in March 2024. Relevant technologies were identified through a process of screening, data extraction and synthesis. A total of 81 unique neurotechnologies were identified, with 23 relating to mental health, 31 to healthy ageing, and 42 to physical disability. A total of 79% percent did not yet have FDA approval and 77.4% were at earlier stages of development (pilot/feasibility studies), with 22.6% at pivotal or post-market stages. Digital elements were common features of the technologies, including software, apps, and connectivity to other devices; however, there were only three technologies with an identifiable AI component. A complex regulatory landscape and unique ethical and safety concerns associated with neurotechnology could pose challenges to innovators, though the emerging nature of the field also provides an opportunity to pre-emptively address potential issues.

## 1. Introduction

Neurotechnology, defined as a health technology (medical device, digital or diagnostic) that enables a direct connection of technical components with the nervous system, is a rapidly emerging field [1]. By 2026, the neurotechnology market is estimated to be worth GBP 14 billion [2].

The ageing demographic and the globalisation of unhealthy lifestyles are expected to lead to a sharp rise in mental and neurological disorders [3]. For health promotion, there is, therefore, a need to address conditions such as these. The potential of neurotechnology in the context of healthcare is vast [4]; six of the ten leading causes of disability worldwide may be targeted by neurotechnology, including Alzheimer’s disease and other dementias, depressive and anxiety disorders, hearing loss, and pain states [5].

In recent years, neuroscience has seen a surge in terms of the number of publications, with brain computer interfaces (BCIs) and deep brain stimulation (DBS) both being prominent areas of neurotechnology research [6]. An increase in the market acceptability of brain stimulation has also been reported [7].

The emerging neurotechnologies (e.g., functional magnetic resonance imaging, neuroprosthetics, invasive and non-invasive modulation, nerve–machine and brain–machine interfaces) provide a wide range of potential therapeutic and non-therapeutic applications, many of which are not yet anticipated. With responsible development and appropriate oversight, this powerful set of technologies may be able to provide major societal benefits [8].

Within the literature, opportunities associated with neurotechnologies have been identified as the ability to be low-risk, affordable, portable, and user-friendly [9]. Other beneficial features include the potential for interactivity and personalisation [9].

However, there are also some possible risks associated with the use of neurotechnology, including a need for better long-term safety and efficacy data, as highlighted by numerous studies [7,9,10], which could lead to ineffective or even harmful treatments. Despite its potential, a review by Levett et al. of invasive BCIs for spinal cord injury also reported unsuitability for home use due to unsuitable and inconvenient set ups, along with long time and specific training requirements [11]. Such factors could act as barriers in the adoption of some neurotechnologies.

Many neurotechnologies can be classed as having elements of digital health technologies, such as wearable devices and apps [12]. With these may come additional concerns including data security and privacy [2,9]. Mitsea et al. also noted concerns over the use of artificial intelligence (AI) algorithms in technologies; biased or inaccurate results from lack of context and differentiation of reliable sources present a risk regarding the safety of AI decision making [9]. It is, therefore, important to ensure that the development and regulation of neurotechnologies is conducted ethically and responsibly and backed up with clinical data.

With the potential of neurotechnology to promote health in terms of several prominent diseases, an awareness of the currently available and upcoming technologies could be beneficial to a variety of stakeholders such as healthcare professionals, regulatory bodies, and patients. Currently, the range of neurotechnologies in use and development is not known, presenting difficulties in knowing both the available treatments and the progress of potential new options.

The aim of this horizon scan was to provide an overview of the neurotechnologies in development and those recently approved, as well as describing their characteristics, including digital health technology elements. The scope of this work covers three key research areas, encompassing conditions which may be promising targets for neurotechnology: mental health, healthy ageing, and physical disability.

## 2. Materials and Methods

A search strategy, which was developed by an Information Specialist and peer-reviewed by a second Information Specialist, was formulated, translated, and performed within each data source. The search strategy combined key words, synonyms, and controlled vocabulary terms (such as MeSH) where indexing was allowed. The full search strategy is included in Appendix A (Table A1, Table A2, Table A3, Table A4, Table A5, Table A6 and Table A7).

A range of sources were searched for clinical trials, conference abstracts, journal articles, and news articles related to neurotechnology. Three clinical trials registries were searched: ClinicalTrials.gov, World Health Organisation (WHO) International Clinical Trials Registry Platform (ICTRP), and Cochrane Register of Controlled Trials (CENTRAL). For ClinicalTrials.gov and WHO ICTRP, no limitations were placed on the searches. For Cochrane CENTRAL, the search was restricted to trials within the last two years. IEEEXplore was searched for conferences and filtered to 2022 to 2024. The Nature journal collection, including Nature, Nature Aging, Nature Biomedical Engineering, Nature Nanotechnology, Nature Neuroscience, Nature Reviews Neurology, and Nature Reviews Neuroscience, was searched in Embase (OVID) and restricted from 2023 to March 2024. GoogleNews and medical technology (MedTech) news websites, including MedTech News, MedTech Dive, Medical Device Network, MedTech Insight, were searched for articles published between 1 January 2023 and 7 March 2024; the oldest relevant article returned from GoogleNews was published in June 2023. GoogleNews was searched using an in-house SCANAR (Search Companion for Advanced News Articles Retrieval) tool [13] which accesses the GoogleNews application programming interface (API), and the MedTech news was searched by utilising a Python 3.12 tool (Selenium) to automate web browsers to extract article elements. Both tools are currently being developed by the Innovation Observatory. The rationale for the confinement of dates within the conference abstracts, journal articles, and news searches was to target the search for the most recent innovations. Searches were performed in March 2024, and deduplicated and combined prior to screening.

The records which the search identified were screened for inclusion against the criteria given in Table 1.

All identified articles were screened for eligibility against the selection criteria. The screening was performed in two stages: title and abstract, followed by full text. The title and abstract screening process was piloted with 100 records being blindly reviewed by five reviewers, with any conflicts resolved through discussion. Records were then split between the five reviewers and single screened at each stage. A second screener resolved inclusion queries.

The following data fields were extracted where possible for all the included records: source; author; sponsor; title; publication year; trial completion year; type of publication; study location; research area; condition; manufacturer; technology name; type of technology; type of device; invasive; AI component; intended setting; stage of treatment; stage of development; FDA approval.

The Devices@FDA database was searched for the technology name and/or manufacturer to determine whether the technology had received FDA approval. The stage of development was determined based on the purpose and/or approximate number of subjects of the latest trial (Table 2).

## 3. Results

### 3.1. Search Results

**Figure 1:**
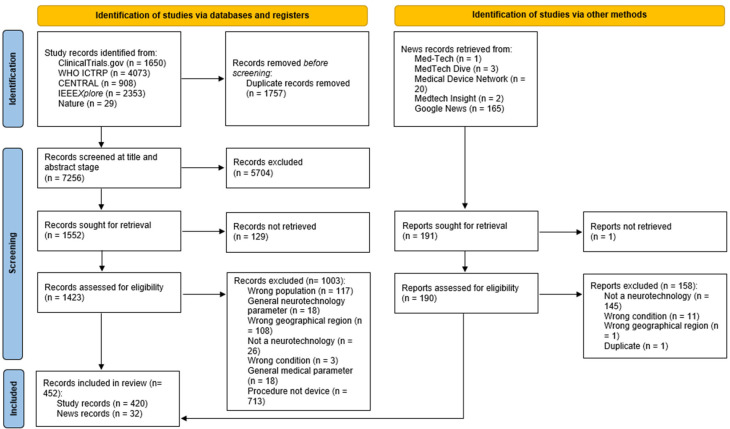
PRISMA flow diagram of identified records.

A total of 7256 records were identified from the search of databases and clinical trial registries after deduplication. Of these, 1423 were brought forward from title and abstract screening to full text screening, following which 430 were included. Additionally, from 190 news articles which were screened, 32 were included. Therefore, in total, 452 records were included for data extraction. The PRISMA flow diagram of identified records is given in Figure 1.

### 3.2. Overview of Identified Technologies

From the included records on which data extraction was performed, 81 unique neurotechnologies meeting the inclusion criteria were identified. For each technology, information was extracted/determined for the following characteristics: FDA approval status; indicated condition(s); type of technology; type of device; invasiveness; presence of an AI component; intended setting; place in care pathway; and digital health technology category. Analysis is presented on each of these categories in the following sections. Additionally, information was collected from clinical trial records associated with each technology. For each condition indicated for a technology, the year of trial (or latest trial if multiple trials existed) was recorded. The development stage was also determined based on clinical trial details using the criteria previously described in Table 2. Full details of each of the identified technologies are included in Table 3 and Table 4, containing those with and without FDA approval, respectively. Where the only source for a technology was a journal article, conference paper, or news (i.e., there was no linked clinical trial identified), this is indicated.

**Figure 2:**
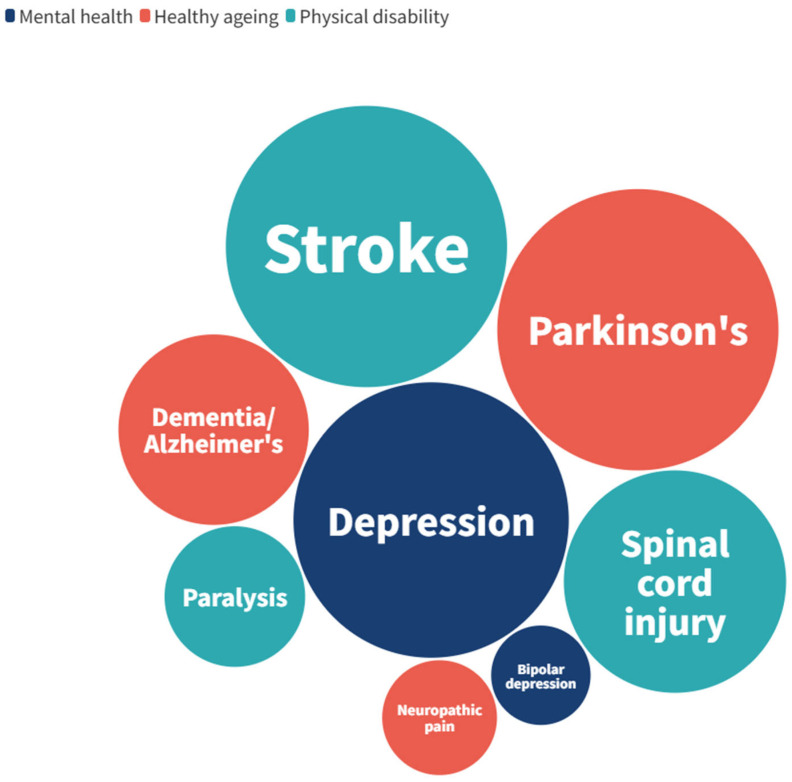
Number of technologies identified for each of the included conditions and research areas.

A total of 81 unique neurotechnologies were identified, with 23 relating to mental health, 31 to healthy ageing, and 42 to physical disability. Of these technologies, there were six which related to two of the three research areas, and five which related to all three research areas. Figure 2 shows a visual representation of the frequency of each condition within each of the research areas. The most commonly indicated conditions were Parkinson’s disease, stroke, and depression. There were no technologies identified for anxiety, personality disorders, or sensory impairment.

A full breakdown of the number of technologies indicated for each of the included conditions is given in Table 5. Where specified, the specific indications are given; bipolar depression accounted for all three technologies indicated for bipolar disorder, and dementia/Alzheimer’s disease could be broken down into dementia (*n* = 1) and Alzheimer’s disease (*n* = 10).

**Figure 3:**
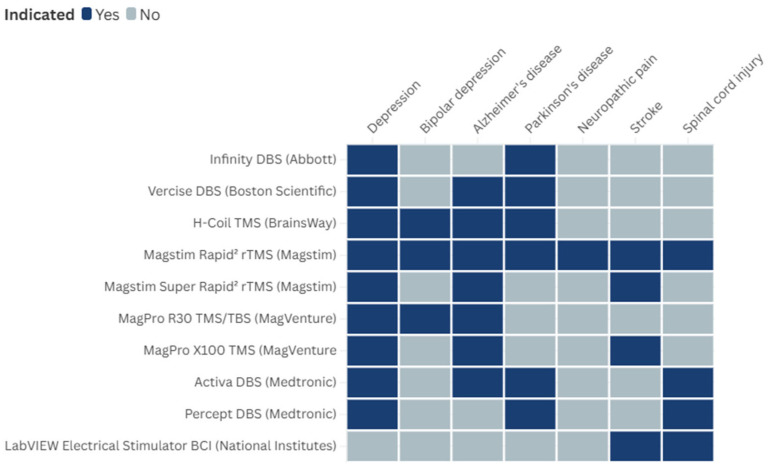
Overlapping of technologies indicated for multiple conditions.

Although most of the identified technologies were indicated for a single included condition, 10 were indicated for more than one. Figure 3 illustrates the spread across the multiple conditions. As the figure shows, there was overlapping of technologies indicated for multiple conditions. For instance, DBS is used to treat depression, Alzheimer’s disease, Parkinson’s disease, and spinal cord injury, while transcranial magnetic stimulation is used to treat depression, bipolar depression, Alzheimer’s disease, Parkinson’s disease, neuropathic pain, stroke, and spinal cord injury, and BCIs are used to treat stroke and spinal cord injury. Each of these technologies showcases the versatility and potential of neurotechnological interventions across various neurological and mental conditions.

**Figure 4:**
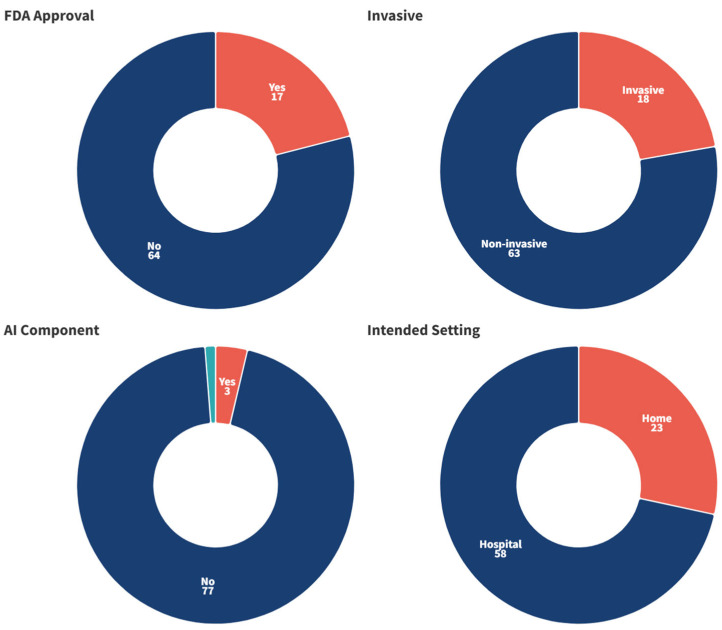
Characteristics of the identified technologies. Reported in terms of the number of technologies.

Figure 4 gives an overview of some of the characteristics of the identified technologies: FDA approval status (yes/no); invasiveness (invasive/non-invasive); presence of an AI component (yes/no/unclear); and intended setting (home/hospital). According to the Devices@FDA database, less than one-quarter (*n* = 17, 21.0%) had received FDA approval. The majority (*n* = 63, 77.8%) were non-invasive technologies; non-invasive neurotechnologies are devices or techniques that interact with the brain without the need for surgical procedures. There were only three (3.7%) technologies for which an AI component was identified, as well as one (1.2%) which was unclear. Most (*n* = 58, 71.6%) of the technologies were intended for a hospital setting, with the remaining 23 (28.4%) being for home use.

**Figure 5:**
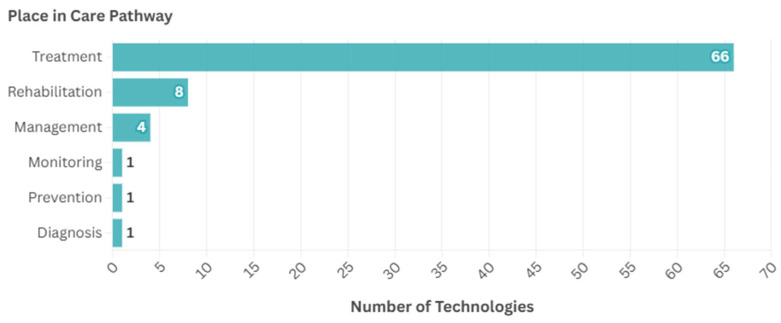
Place in care pathway for the identified technologies.

In terms of place in the care pathway, the most common stage was treatment (*n* = 66, 81.5%), followed by rehabilitation (*n* = 8, 9.9%), with fewer intended for other uses: management (*n* = 4, 4.9%), monitoring (*n* = 1, 1.2%), prevention (*n* = 1, 1.2%), and diagnosis (*n* = 1, 1.2%), as shown in Figure 5.

**Figure 6:**
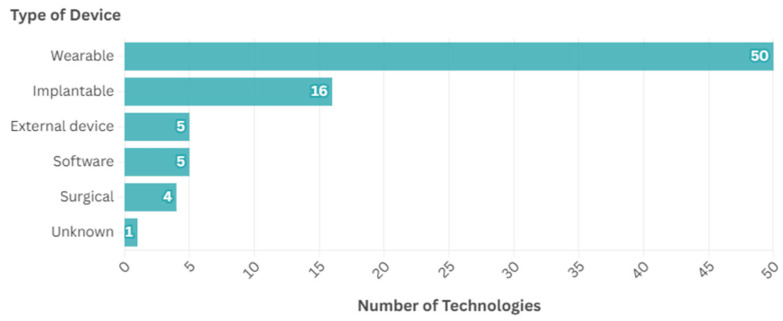
Types of devices identified.

Several types of devices were identified, as given by Figure 6. The most common type of device identified were wearables (*n* = 50, 61.7%), followed by implantable devices (*n* = 16, 19.8%), with fewer falling into other categories: external devices (*n* = 5, 6.2%), software (*n* = 5, 6.2%), surgical (*n* = 4, 4.9%), and one (1.2%) unknown.

**Figure 7:**
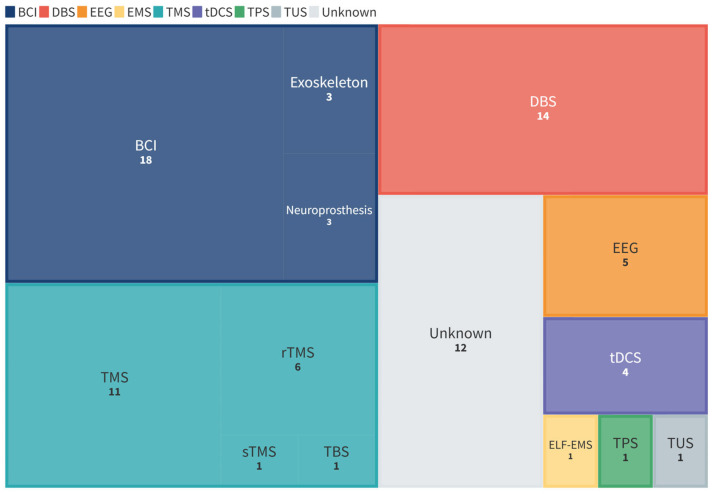
Types of technologies identified. BCI = brain computer interface; DBS = deep brain stimulation; EEG = electroencephalography; ELF-EMS = extremely low- frequency electromagnetic stimulation; tDCS = transcranial direct current stimulation; TPS = transcranial pulse stimulation; TUS = transcranial ultrasound stimulation; TMS = transcranial magnetic stimulation; rTMS = repetitive TMS (transcranial magnetic stimulation; sTMS = single-pulse transcranial magnetic stimulation; TBS = theta-burst stimulation.

The types of technologies were classified according to the following broad categories: brain computer interface (BCI); deep brain stimulation (DBS); electroencephalography (EEG); electromagnetic stimulation (EMS); transcranial magnetic stimulation (TMS); transcranial direct current stimulation (tDCS); transcranial pulse stimulation (TPS); transcranial ultrasound stimulation (TUS). TMS was then further broken down to include repetitive TMS (rTMS), single-pulse TMS (sTMS), and theta-burst stimulation (TBS). EMS included extremely low-frequency electromagnetic stimulation (ELF-EMS). Figure 7 shows the breakdown by types of technologies. The majority of the technologies fell into the categories of BCIs, TMS, and DBS. Specifically, 24 (29.6%) were BCIs, 19 (23.5%) were TMS, 14 (17.3%) were DBS, 5 (6.2%) were EEG, 4 (4.9%) were tDCS, 1 (1.2%) was EMS, 1 (1.2%) was TPS, 1 (1.2%) was TUS, and 12 (14.8%) were unknown.

**Figure 8:**
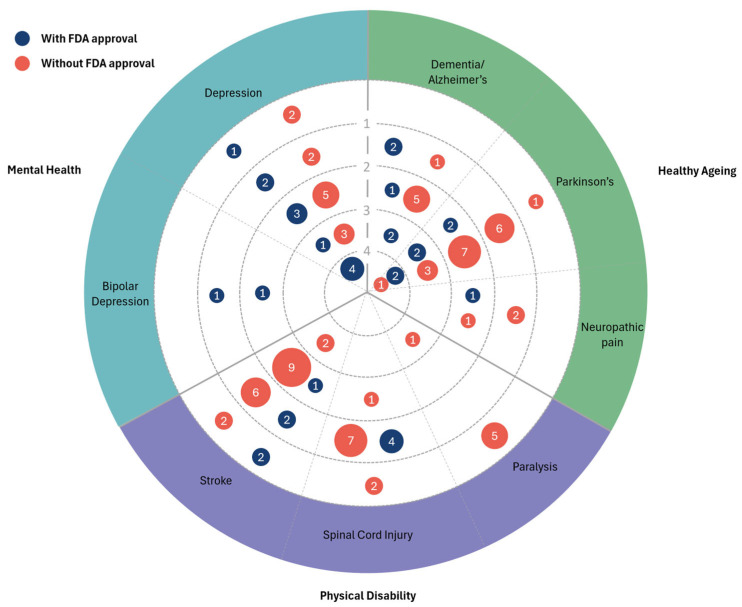
Technology radar visualising the stages of development of the identified technologies for each condition. The size of the bubbles and values within refer to the number of technologies identified at each stage for each condition, and the colours correspond to whether the technology has FDA approval.

A technology radar (Figure 8) was developed to visualise the stage of development of the identified technologies for each of the included conditions. There was no stage of development associated with 15 (18.5%) of the technologies due to no linked clinical trials being found for these; these are shown in the outermost ring of the radar. There were 35 (43.2%) technologies at stage one of development (pilot/early feasibility), 37 (45.7%) at stage two (traditional feasibility), 15 (18.5%) at stage three (pivotal), and 7 at stage four (post-market) (4.9%). The radar shows the number of technologies both with and without FDA approval at each stage of development for the different conditions. From this, it can be seen that most of the technologies were at earlier stages of development, with fewer at pivotal or post-market stages.

### 3.3. Further Analysis of Digital Neurotechnologies

**Figure 9:**
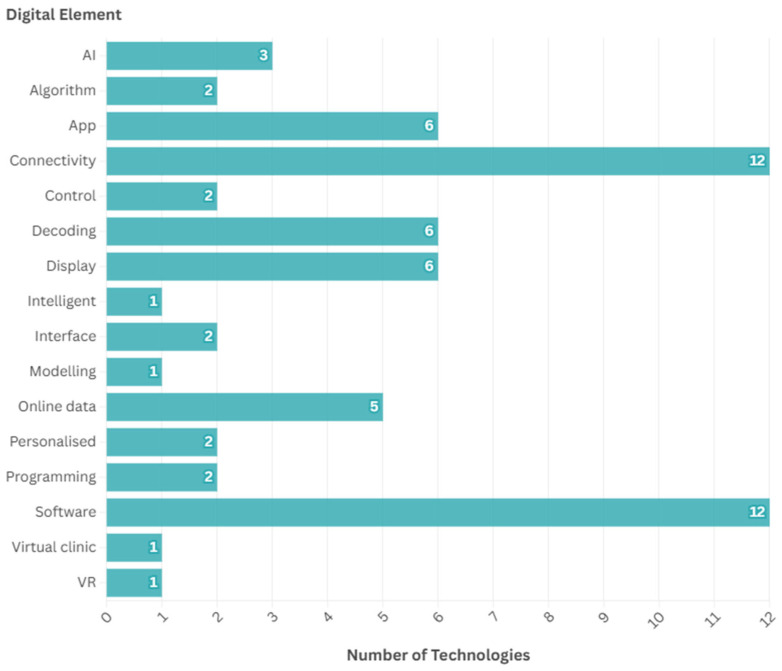
Digital elements identified. AI = artificial intelligence. VR = virtual reality.

Of the 81 unique neurotechnologies identified, information was not found on any specific digital elements for 23. This was due to either a lack of information on the technology (e.g., for technologies at an early stage of development), or if a specific digital element could not be found. The digital elements identified for the remaining 58 technologies are given in Figure 9; 5 of the technologies had more than one digital element identified. Common elements included software and connectivity. Twelve technologies had associated software, as well as six which specifically had an app, and twelve had an element of connectivity (to devices such as a computer, phone, or keyboard).

There are five main categories of patient-facing digital health technologies, as defined by the Digital Therapeutics Alliance, which can be differentiated by four key factors: label claims, evidence requirements, and regulatory implications. These criteria are given in Table 6 and was used to determine the category of digital health technology for the neurotechnologies with digital elements identified [16].

**Figure 10:**
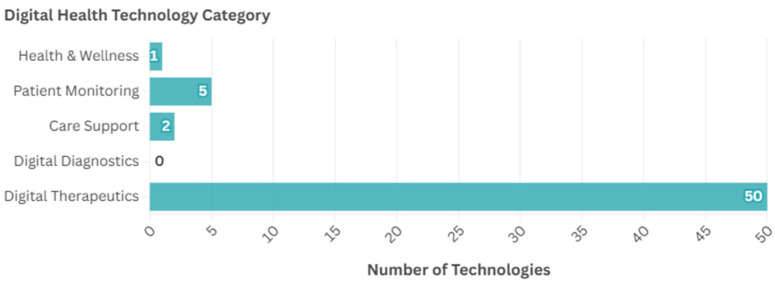
Number of neurotechnologies with each category of digital health technology elements.

Figure 10 shows the proportion of the neurotechnologies with digital elements that were classed as each of the different digital health technology categories. The majority (*n* = 50, 86.2%) of the 58 neurotechnologies with digital elements identified in this horizon scan were classed as therapeutics, with the technology claiming to treat or alleviate a medical condition (e.g., to deliver stimulation). There were relatively few classed as health and wellness (e.g., for general wellness) (*n* = 1, 1.7%), patient monitoring (e.g., for recording brain activity) (*n* = 5, 8.6%), or care support (e.g., virtual clinic) (*n* = 2, 3.4%), and no diagnostics.

## 4. Discussion

This horizon scan explored the innovation pipeline for neurotechnology across mental health, healthy ageing, and physical disability, to provide an overview of the currently available and emerging technologies.

Our scan showed that technologies for Parkinson’s disease, stroke, and depression were most common. Stroke and depression are amongst the ten leading causes of disability globally, and the ageing population means that the prevalence of age-related conditions such as Parkinson’s disease is expected to increase [5,16,17]. Additionally, mental health is an increasing concern for public health that has also been linked to the ageing population of developed countries [4]. The relatively high proportion of technologies identified in this horizon scan targeting these conditions may, therefore, reflect these public health priorities. However, it should also be noted that the scope of this horizon scan covered only select conditions within the three research areas.

There is also potential for the same neurotechnology to be applied to multiple conditions; ten of the technologies identified were targeting more than one of the included conditions, including one (Magstim Rapid^2^, Magstim) which targeted all seven for which technologies were identified (depression, bipolar depression, Parkinson’s disease, Alzheimer’s disease, neuropathic pain, stroke, and spinal cord injury). This illustrates the potential that neurotechnology has within the areas of mental health, healthy ageing, and physical disability.

Despite regulatory approval being crucial for new medical devices [18], there were relatively few technologies identified with FDA approval. Aligned with this, most of the technologies were at earlier stages of development, with few at post-market, which could reflect the length of the process needed to gather the clinical evidence required for regulatory approval. Several barriers related to commercialisation of neurotechnologies have also been previously reported following interviews with stakeholders, including difficulty in navigating the regulatory pathways, limited capacity of regulators, and clinical evidence requirements [19]. These challenges could impact the innovation of neurotechnologies, particularly at the later stages, when technologies near market readiness.

Within this horizon scan, the majority of the identified technologies were non-invasive. The classification system by risk for medical devices means that non-invasive neurotechnologies generally have slightly less stringent regulatory requirements—including the level of clinical evidence required—than invasive neurotechnologies [20,21]. While regulatory oversight has been highlighted as a key area of consideration for neurotechnologies [22], potential implications for innovation have been argued [23]. However, this goes beyond the scope of the present work.

Three technologies identified in this scan had an AI component, and a further two specified incorporating personalised treatment, for example, monitoring brain signals to then inform the location of the stimulation given. In this way, neurotechnology devices may be more targeted to an individual. The three technologies identified as having an AI component were all wearables, though each was targeting a different condition (Parkinson’s disease, stroke, and spinal cord injury). Although a trend in integration of AI with neurotechnology has been suggested within the literature, with the potential for AI to enhance the functionality and usability of neurotechnologies [24,25], the identified technologies with an AI component within this horizon scan were all at earlier stages of development (one at stage one, one at stage two, and one without an ascertained stage). AI has the potential to enhance the functionality and user experience of neurotechnologies and therefore, there may be more neurotechnologies facilitated by AI seen in the future, as both fields continue to advance.

In terms of the types of devices within this scan, the most common type was wearables, of which we identified and included 50 such technologies covering different purposes from delivering stimulation to monitoring symptoms, with some performing multiple functions concurrently. Technologies such as these may enable home-based treatments—or treatments via virtual wards, also known as hospital at home, an initiative which has seen rapid growth within the NHS across England—which in turn can provide equity of access and ease of use for patients [26]. Therefore, incorporating digital elements such as apps to allow patients to control the technology may help to facilitate meeting these needs.

The five categories of patient-facing digital health technologies, as detailed previously in Table 6, can be differentiated according to four key considerations: label claims, intervention delivery, evidence requirements, and regulatory implications. For the neurotechnologies identified in this review, these could be analysed in terms of their digital elements. The vast majority of the neurotechnologies with digital elements identified were therapeutics. Since these technologies deliver an intervention to treat or alleviate a medical condition, they are regulated and must meet evidence requirements for efficacy claims. It is to be expected that this horizon scan would predominantly identify these types of technologies, since clinical trial registries were the primary information source searched, which would typically contain regulated technologies, which require clinical evidence. In contrast, health and wellness technologies, which are not regulated and do not have evidence requirements, would be less likely to be found. It is therefore unsurprising that this horizon scan identified only one such technology—Elemind’s first product, which is positioned as a general wellness device, and therefore not subject to regulation by the FDA—with this being from news sources [27]. There were also relatively few technologies identified which may require evidence and regulatory approval (i.e., patient monitoring and care support technologies). Specifically, there were five technologies with digital elements for patient monitoring, and two for care support. There were also some technologies identified which had elements of digital patient monitoring or care support, for example, a technology consisting of a device which delivers stimulation along with an app to track symptoms. In cases such as this, where the primary claim of the technology was to deliver a medical intervention, the technology was classed as a therapeutic. Although only 8 of the 50 neurotechnologies with digital elements identified were not classed as therapeutics and therefore not necessarily subject to the same stringent level of regulatory oversight and evidence requirements, it is likely that there are more of these technologies either in development or already on the market, that were not identified in this scan.

Recognising the varied characteristics of the technologies identified in this horizon scan—including invasiveness, AI components, and other digital elements—ethical concerns including safety, data security, and privacy are inherent with the innovation of neurotechnologies [9,24]. Data privacy and security have previously been highlighted as important considerations for digital health technologies which can result in challenges with the commissioning of these technologies in the UK [25]. Data concerns are particularly relevant to neurotechnologies as some form of data monitoring is common with neurotechnologies, since these data can then be used to inform treatment (e.g., recording brain signals to then inform the stimulation that is administered). Furthermore, five of the technologies from this scan were identified as having online (cloud-based) data storage. Such ethical considerations that are associated with digital health technologies (ensuring data privacy and security) are therefore also important for neurotechnologies, many of which have digital elements. In terms of safety concerns, a lack of long-term research on the effectiveness of neurotechnologies creates the potential for ineffective or even harmful treatments to be introduced. However, there is also an opportunity for those involved in neurotechnology innovation to take pre-emptive action prior to any potential safety or ethical issues arising, given the emerging nature of the field [25]. Consideration of the issues observed, including ensuring data security and privacy, and long-term safety and efficacy results, could contribute to the responsible innovation of neurotechnologies to ultimately benefit patients.

This horizon scan sought to provide an overview of the neurotechnologies in development and those approved by the FDA as of June 2024 for mental health, healthy ageing, and physical disabilities. A comprehensive search was formulated by combining multiple information sources of which relevant signals of research may be found. However, there were some limitations. The scope of the horizon scan encompassed select conditions—chosen on the basis of being promising targets for neurotechnology—and so does not include neurotechnologies for any other indications. There are also different possible definitions of neurotechnology, of which one was used for this work, which could influence which technologies are deemed relevant at the screening stage. Finally, challenges in finding data meant that only the FDA database was used for determining whether technologies had regulatory approval. This was due to difficulty in accessing approval records from other regulatory bodies: the UK Medicines and Healthcare Products Regulatory Agency (MRHA), the Public Access Registration Database (PARD), and the European Database on Medical Devices (EUDAMED). Nevertheless, this horizon scan provides an up-to-date insight on the innovation pipeline for neurotechnologies in these three areas of interest.

## 5. Conclusions

In conclusion, this horizon scan provides valuable insights into the neurotechnology innovation pipeline to promote health in terms of addressing some of the leading causes of disability globally. The emerging nature of neurotechnology was highlighted, with most of the technologies identified being at earlier stages of development. Many of the technologies had various digital elements linked; as the neurotechnology field continues to advance and further converge with digital health technologies, there is potential for these neurotechnologies to progress into pivotal stages and become available in the near future. Whilst acknowledging the need for ensuring safety and efficacy through clinical and regulatory processes, the continued development of these technologies could enable patients to access new treatment options.

## Figures and Tables

**Table 1:** Inclusion criteria.

Characteristic	Criteria
MedTech	A regulated medical device, digital health technology, or diagnostic, as defined by EU Regulation 2017/745 [14] and 2017/746 [15]
Neurotechnology	Defined as enabling a direct connection of technical components with the nervous system
Condition(s)	Mental health: anxiety; bipolar disorder; depression; personality disorders
	Healthy ageing: dementia/Alzheimer’s disease; Parkinson’s disease; sensory impairment; neuropathic pain
	Physical disability: stroke; spinal cord injury (or paralysis where it could be due to stroke or spinal cord injury)
Population	Adults (aged 18 years and older)
Language	English
Trial location	UK, Europe, North America, Latin America, Middle East, Africa, Australia, or multiple sites

**Table 2:** Stages of development.

Stage of Development	Subjects	Purpose
1: Pilot/early feasibility/first-in-human	10–30	Collect data on preliminary safety and device performance in humans
2: Traditional feasibility	20–30	Assess safety and efficacy of final or near-final device in humans
3: Pivotal	100 s	Confirm clinical efficacy and safety
4: Post-market	1000 s	Monitor long-term effectiveness, safety and usage in the general population

**Table 3:** Identified technologies with FDA approval.

Technology (Manufacturer)	Number of Trials (Date Range of Trial) Condition (Year of Latest Trial): Development Stage	Type of Technology	Type of Device	Invasive?	AI Component?	Intended Setting	Place in Pathway	Digital Health Technology Category
Neuro-Omega System (Alpha Omega Engineering)	Parkinson’s disease (2017): Stage 3	DBS	Surgical			Hospital	Treatment	Digital therapeutics
NeuroPort Array (Blackrock Neurotech)	Spinal cord injury (2013): Stage 1	BCI	Implantable			Hospital	Treatment	
Vercise (Boston Scientific)	12 trials (2010–2025) Parkinson’s disease (2020): Stage 3 Alzheimer’s disease (2025): Stage 1 Depression: (2018): Stage 2	DBS	Implantable			Hospital	Treatment	
EGI Geodesic N400 System (Electrical Geodesics)	Stroke *	EEG	Wearable			Hospital	Rehabilitation	Patient monitoring
Rehastim 2 (HASOMED)	Stroke ^†^	BCI	External device			Home	Treatment	Digital therapeutics
SAINT (Magnus Medical)	2 trials (2023–2024) Major depressive disorder (2023): Stage 4	TMS	Wearable			Home	Treatment	Digital therapeutics
Magstim 200^2^ (Magstim)	2 trials (2013–2010) Parkinson’s disease (2010): Stage 2	rTMS	Wearable			Hospital	Treatment	
Magstim Rapid^2^ (Magstim)	16 trials (2007–2023) Stroke (2023): Stage 2 Spinal cord injury (2016): Stage 1 Alzheimer’s disease (2012): Stage 2 Parkinson’s disease (2015): Stage 2 Depression (2012): Stage 2 Neuropathic pain (2022): Stage 2 Bipolar depression (2012): Stage 1	rTMS	Wearable			Hospital	Treatment	
StimGuide (Magstim)	Major depressive disorder (2019): Stage 2	TMS	Wearable			Hospital	Treatment	Digital therapeutics
MagPro R30 (MagVenture)	10 trials (2015–2024) Depression (2024): Stage 3 Bipolar depression (2023): Stage 2 Alzheimer’s disease (2023): Stage 3	TMS/TBS	External device			Hospital	Treatment	
MagPro X100 (MagVenture)	11 trials (2010–2024) Stroke (2010): Stage 1 Depression (2024): Stage 4 Alzheimer’s disease (2023): Stage 3	TMS	External device			Hospital	Treatment	
Activa (Medtronic)	20 trials (2005–2017) Depression (2014): Stage 1 Spinal cord injury (2015): Stage 1 Alzheimer’s disease (2012): Stage 1 Parkinson’s disease (2017): Stage 4	DBS	Implantable			Hospital	Treatment	
Percept (Medtronic)	8 trials (2013–2023) Parkinson’s disease (2023): Stage 4 Depression (2023): Stage 1 Spinal cord injury (2021): Stage 1	DBS	Implantable			Hospital	Treatment	Digital therapeutics
Relivion (Neurolief)	Major depressive disorder ^††^	Unknown	Wearable			Home	Treatment	Digital therapeutics
NeuroStar TMS (Neuronetics)	4 trials (2011–2020) Major depressive disorder (2020): Stage 4	TMS	Wearable			Hospital	Treatment	Digital therapeutics
NeuroPace RNS System (NeuroPace)	1 trial (2019) Depression (2019): Stage 4	DBS	Surgical			Hospital	Treatment	Digital therapeutics
eXimia NBS System (NeuroPace)	1 trial (2009) Stroke (2009): Stage 1	TMS	Wearable			Hospital	Rehabilitation	Digital therapeutics

Key: * conference paper; ^†^ journal article; ^††^ news.

**Table 4:** Identified technologies without FDA approval.

Technology (Manufacturer)	Number of Trials (Date Range of Trial) Condition (Year of Latest Trial): Development Stage	Type of Technology	Type of Device	Invasive?	AI Component?	Intended Setting	Place in Pathway	Digital Health Technology Category
LiveAmp Mobile Amplifier + XoMotion (Human in Motion Robotics; BrainProducts)	Paralysis ^†^	BCI	Wearable			Home	Treatment	Digital therapeutics
RECOM	Stroke (2023): Stage 1	BCI	Wearable			Hospital	Treatment	
Infinity (Abbott)	3 trials (2019) Parkinson’s disease (2019): Stage 4 Depression (2019): Stage 1	DBS	Implantable	✔		Hospital	Treatment	Digital therapeutics
Neurosphere Virtual Clinic (Abbott)	Parkinson’s disease (2022): Stage 2	DBS	Software			Home	Treatment	Care support
directSTIM System (Aleva Neurotherapeutics SA)	2 trials (2012–2021) Parkinson’s disease (2021) Stage 2	DBS	Implantable	✔		Hospital	Treatment	Digital therapeutics
Axem Home (Axem Neurotechnology)	Stroke (2023): Stage 1	Near-infrared spectroscopy headband	Wearable			Home	Rehabilitation	Care support
Picostim DBS System (Bioinduction)	Parkinson’s disease (2020): Stage 1	DBS	Implantable	✔		Hospital	Treatment	Digital therapeutics
Combined EEG and fNIRS Device (BioSignal Group; NIRx Medizintechnik GmbH)	Stroke (2017): Stage 1	EEG	Wearable			Hospital	Diagnosis	
Move Again (Blackrock Neurotech)	Paralysis ^††^	BCI	Implantable	✔		Hospital	Treatment	Digital therapeutics
BrainGate Neural Interface System (BrainGate)	Spinal cord injury (2009): Stage 1	BCI	Implantable			Hospital	Treatment	Digital therapeutics
BrainQ (BrainQ)	2 trials (2018–2019) Spinal cord injury (2019): Stage 1	EMS	Wearable		✔	Home	Treatment	Digital therapeutics
H-Coil (BrainsWay)	9 trials (2006–2023) Parkinson’s disease (2014): Stage 1 Alzheimer’s disease (2010): Stage 2 Depression (2009): Stage 3 Bipolar depression (2014): Stage 2	TMS	Wearable			Hospital	Treatment	Digital therapeutics
ECoG Measuring Implant (Clinatec)	Spinal cord injury (2015): Stage 1	BCI	Wearable			Hospital	Treatment	Digital therapeutics
Headband (Elemind)	Parkinson’s disease ^††^	EEG	Wearable		✔	Home	Treatment	Health and wellness
The Flow (Flow Neuroscience)	Depression *	tDCS	Wearable			Home	Treatment	Digital therapeutics
recoveriX (g.tec)	Stroke (2019): Stage 1	BCI	Wearable			Hospital	Treatment	Digital therapeutics
Gondola AMPS Device (Gondola Medical Technologies SA)	Parkinson’s disease (2021): Stage 2	DBS	External device			Home	Treatment	
LG-7500 Digital Muscle Stimulator (LGMedSupply)	Stroke (2010): Stage 3	BCI	Wearable			Hospital	Treatment	
rTMS Device (Madinatab Iran)	Parkinson’s disease (2023): Stage 1	rTMS	Wearable			Hospital	Rehabilitation	
Magstim Super Rapid^2^ (Magstim)	7 trials (1996–2023) Depression (2015): Stage 3 Alzheimer’s disease (2023): Stage 2 Stroke (2015): Stage 2	rTMS	Wearable			Hospital	Treatment	Digital therapeutics
Magstim Rapid^2^ Plus^1^ (Magstim)	Stroke (2023): Stage 2	rTMS	Wearable			Hospital	Treatment	Digital therapeutics
Cool Coil (MagVenture)	3 trials (2013–2020) Depression (2020): Stage 3	TMS	Wearable			Hospital	Treatment	
GENUS Device (Massachusetts Institute of Technology)	3 trials (2019–2025) Alzheimer’s disease (2025): Stage 1 Parkinson’s disease (2019): Stage 2	Gamma frequency stimulation	Wearable			Home	Prevention	
SMARTING Device (mBrainTrain)	Neuropathic pain after spinal cord injury (2020): Stage 1	EEG	Wearable			Hospital	Treatment	Patient monitoring
MAHI EXO-II (Mechatronics and Haptic Interfaces Lab)	Stroke (2013): Stage 1	BCI	Wearable			Hospital	Treatment	Digital therapeutics
DOT Microstimulator (Motif Neurotech)	Depression ^††^	Unknown	Implantable	✔		Hospital	Treatment	
BrainSense EEG Headset	Spinal cord injury *	Unknown	Wearable		?	Home	Treatment	Patient monitoring
Networked Neuroprosthetic System (National Institute of Neurological Disorders and Stroke)	Spinal cord injury (2014): Stage 2	Neuroprosthetic	Surgical	✔		Hospital	Rehabilitation	Digital therapeutics
Telepathy (Neuralink)	Quadriplegia ^††^	BCI	Implantable	✔		Hospital	Treatment	Digital therapeutics
Exobots System (Neurobots)	Stroke (2019): Stage 2	BCI	Wearable			Hospital	Treatment	Digital therapeutics
DC Stimulator Plus (NeuroCare)	4 trials (2016–2021) Fibromyalgia depression and neuropathic pain (2016): Stage 2 Parkinson’s disease (2021): Stage 3 Alzheimer’s disease (2021): Stage 2	TMS	Wearable			Hospital	Treatment	Digital therapeutics
Power Mag (NeuroCare)	1 trial (2022) Alzheimer’s disease (2020): Stage 2	rTMS	Wearable			Hospital	Treatment	Digital therapeutics
Starstim tDCS (Neuroelectrics)	1 trial (2021) Parkinson’s disease (2021): Stage 1	tDCS	Wearable			Hospital	Treatment	Patient monitoring
NeuroFUS Device (NeuroFUS)	1 trial (2023) Parkinson’s disease (2023): Stage 1	TUS	Wearable			Hospital	Treatment	
IpsiHand (Neurolotions)	3 trials (2012–2023) Stroke (2023): Stage: 3	BCI	Wearable			Home	Rehabilitation	Digital therapeutics
Neurow System (NeuroRehabLab)	1 trial (2021) Stroke (2021): Stage 2	BCI	Wearable		✔	Hospital	Treatment	Digital therapeutics
MS and MSD Equipment (Neurosoft)	1 trial (2019) Parkinson’s disease (2019): Stage 2	TMS	Wearable			Hospital	Treatment	Digital therapeutics
AlphaDBS (Newronika)	4 trials (2017–2022) Parkinson’s disease (2022): Stage 3	DBS	Implantable	✔		Hospital	Treatment	Digital therapeutics
LabVIEW Electrical Stimulator (National Institutes)	Stroke/Spinal cord injury *	BCI	Software			Home	Treatment	Digital therapeutics
NeuroCognitive Communicator (Ottawa Hospital Research Institute)	1 trial (2019) Stroke (2019): Stage 1	BCI	Wearable			Home	Management	Digital therapeutics
PD-Monitor (PD Neurotechnology)	1 trial (2022) Parkinson’s disease (2022): Stage 3	Monitoring device	Wearable			Home	Monitoring	Patient monitoring
Layer 7 Cortical Interface (Precision Neuroscience)	1 trial (2023) Severe paralysis (2023): Stage 3	Unknown	Implantable	✔		Hospital	Treatment	Digital therapeutics
Dynamic Environment-Based Visual Interface System	1 trial (2022) Paralysis (2022): Stage 1	Unknown	Software			Home	Treatment	Digital therapeutics
Sapiens (Sapiens Steering Brain Stimulation BV)	1 trial (2012) Parkinson’s disease (2012): Stage 1	DBS	Surgical	✔		Hospital	Treatment	Digital therapeutics
M4P-System (SensorStim Neurotechnology GmbH)	1 trial (2021) Parkinson’s disease (2021): Stage 2	Unknown	Wearable			Hospital	Treatment	
TMS Cap (Seraya Medical Systems)	1 trial (2016) Stroke (2016): Stage 2	TMS	Wearable			Hospital	Treatment	
BCI (Smart Wheelchair)	Paralysis *							
Sooma tDCS (Sooma)	Depression (2019): Stage 2	tDCS	Wearable			Home	Treatment	Digital therapeutics
tDCS Mini-Clinical Trials System (Soterix)	Depression (2018): Stage 2	tDCS	Wearable			Hospital	Treatment	Digital therapeutics
NEUROLITH TPS (Storz Medical AG)	Dementia (2024): Stage 2	TPS	External device			Hospital	Treatment	Digital therapeutics
Synchron Switch (Synchron)	Paralysis ^††^	BCI	Implantable	✔		Hospital	Treatment	Digital therapeutics
TyroTherapy (Tyromotion)	Stroke (2022): Stage 2	Unknown	Wearable			Hospital	Treatment	Digital therapeutics
Tele-REINVENT (University of Southern California)	Stroke (2021): Stage 1	Unknown	Wearable			Home	Treatment	Digital therapeutics
GHOST	Neuropathic pain (2019): Stage 1	BCI	Wearable			Hospital	Treatment	
BCI-NMES	Stroke (2017): Stage 2	Unknown	Implantable			Hospital	Treatment	
NEST-1 NeoSync EEG Synchronised TMS (Wave Neuroscience)	Depression (2011): Stage 2	sTMS	Wearable			Hospital	Treatment	Digital therapeutics
H-Coil (Weizmann Institute of Science)	Depression (2007): Stage 2	TMS	Wearable			Hospital	Treatment	
The Promoter	Stroke (2023): Stage 2	BCI	Unknown			Hospital	Rehabilitation	Digital therapeutics
CereGate Software	Parkinson’s disease (2024): Stage 2	DBI	Software			Hospital	Treatment	Digital therapeutics
NeuroExo	Stroke (2022): Stage 2	BCI-EEG	Wearable			Home	Rehabilitation	Digital therapeutics
Libra Implantable DBS System	2 trials (2013–2016) Depression (2016): Stage 1	DPS	Implantable	✔		Hospital	Treatment	
RoBIK	Spinal cord injury (2018): Stage 1	BCI	Software			Hospital	Management	Digital therapeutics
MoreGrasp	Spinal cord injury (2018): Stage 1	BCI-neuroprosthesis	Wearable			Home	Management	Digital therapeutics
Mind Extender (MindEx)	Spinal cord injury (2024): Stage 1	BCI	Wearable			Home	Management	Digital therapeutics

Key: * conference paper; ^†^ journal article; ^††^ news. “✔” refers to the technology possessing this characteristic; “?” refers to it being unknown whether the technology possesses this characteristic.

**Table 5:** Number of technologies identified for each condition.

Research Area	Condition	Number of Technologies
Mental health	Depression	23
	Anxiety	-
	Bipolar disorder	3 *
	Personality disorders	-
Healthy ageing	Dementia/Alzheimer’s disease	11 ^†^
	Parkinson’s disease	24
	Sensory impairment	-
	Neuropathic pain	4
Physical disability	Stroke	24
	Spinal cord injury	15
	Paralysis	6

* Bipolar depression. ^†^ 1 dementia, 10 Alzheimer’s disease.

**Table 6:** Overview of patient-facing digital health technologies [16].

	Health and Wellness	Patient Monitoring	Care Support	Digital Diagnostics	Digital Therapeutics
Label Claims	No claims to treat, improve, or diagnose	May make non-clinical claim to monitor health data	May make non-clinical claim to manage care	Make clinical claim to diagnose or assess	Make clinical claim to treat or alleviate
Intervention Delivery	Does not deliver medical intervention	Collects health data to inform decision making around medical intervention	Does not deliver medical intervention; may make recommendations	Drives medical intervention through diagnosis or assessment	Delivers medical intervention
Evidence Requirements	No	Yes—non-clinical claims	Yes—non-clinical claims	Yes—diagnostic accuracy	Yes—efficacy claims
Regulatory Implications	No regulatory oversight	Regulatory approval may be required	Regulatory approval may be required	Regulated	Regulated

## Data Availability

The original contributions presented in this study are included in the article/Appendix A. Further inquiries can be directed to the corresponding author.

## Abbreviations

The following abbreviations are used in this manuscript:

AI	Artificial intelligence
API	Application programming interface
BCI	Brain computer interface
CEN-TRAL	Cochrane Register of Controlled Trials
DBS	Deep brain stimulation
EEG	Electroencephalography
ELF-EMS	Extremely low frequency electromagnetic stimulation
EMS	Electromagnetic stimulation
EUDAMED	European Database of Medical Devices
FDA	Food & Drug Administration
HealthTech	Health technology
ICTRP	International Clinical Trials Registry Platform
MedTech	Medical technology
MeSH	Medical Subject Headings
MHRA	Medicines and Healthcare Products Regulatory Agency
NHS	National Health Service
PARD	Public Access Registration Database
PRISMA	Preferred Reporting Items for Systematic Reviews and Meta-Analyses
rTMS	Repetitive transcranial magnetic stimulation
sTMS	Single-pulse transcranial magnetic stimulation
TBS	Theta-burst stimulation
tDCS	Transcranial direct current stimulation
TMS	Transcranial magnetic stimulation
TPS	Transcranial pulse stimulation
TUS	Transcranial ultrasound stimulation
UK	United Kingdom
VR	Virtual reality
WHO	World Health Organization

## Appendix A

The search strategies for each information source are provided in Table A1, Table A2, Table A3, Table A4, Table A5, Table A6 and Table A7.

**Table A1:** ClinicalTrials.gov search strategy.

#	String	Results
1	“deep brain stimulation” OR neuroprosthetic OR photostimulation OR “transcranial magnetic stimulation” OR “transcranial electric stimulation” OR “brain–computer interfaces” OR “brain interface” OR “brain augmentation” OR “brain computer” OR neurotechnology OR cognitive technology OR (brain AND prosthetic)	1650

Search date: 20 March 2024.

**Table A2:** WHO ICTRP search strategy.

#	String	Results
1	“deep brain stimulation” OR neuroprosthetic OR photostimulation OR “transcranial magnetic stimulation” OR “transcranial electric stimulation” OR “brain–computer interfaces” OR brain interface OR brain augmentation OR “brain computer” OR neurotechnology OR cognitive technology OR (brain AND prosthetic)	4073

Search date: 20 March 2024.

**Table A3:** CENTRAL search strategy.

#	String	Results
1	(“neuroprosthetic”):ti,ab,kw	13
2	((brain AND prosthetic)):ti,ab,kw	47
3	(Photostimulation):ti,ab,kw	153
4	(“Brain–computer interfaces”):ti,ab,kw	140
5	(Brain interface):ti,ab,kw	599
6	(Brain augmentation):ti,ab,kw	435
7	(“Brain computer”):ti,ab,kw	401
8	MeSH descriptor: [Brain-Computer Interfaces] this term only	105
9	(Neurotechnology):ti,ab,kw	32
10	(Cognitive technology):ti,ab,kw	2133
11	#1 OR #2 OR #3 OR #4 OR #5 OR #6 OR #7 OR #8 OR #9 OR #10 with Cochrane Library publication date in the last 2 years, in trials	908

Search date: 20 March 2024.

**Table A4:** IEEEXplore search strategy.

#	Search	Results
1	(“Document Title”:“deep brain stimulation” OR “Abstract”:“deep brain stimulation” OR “Document Title”:Neuroprosthetic OR “Abstract”:Neuroprosthetic OR “Document Title”:Photostimulation OR “Abstract”:Photostimulation OR “Document Title”:“Transcranial magnetic stimulation” OR “Abstract”:“Transcranial magnetic stimulation” OR “Document Title”:“Transcranial electric stimulation” OR “Abstract”:“Transcranial electric stimulation” OR “Document Title”:“Brain–computer interfaces” OR “Abstract”:“Brain–computer interfaces” OR “Document Title”:Brain interface OR “Abstract”:Brain interface OR “Document Title”:Brain augmentation OR “Abstract”:Brain augmentation OR “Document Title”:“Brain computer” OR “Abstract”:“Brain computer” OR “Document Title”:Neurotechnology OR “Abstract”:Neurotechnology OR “Document Title”:Cognitive technology OR “Abstract”:Cognitive technology OR “Document Title”:Brain AND prosthetic OR “Abstract”:Brain AND prosthetic)	20,937
2	Filters Applied: 2022–2024	3403
3	Filters Applied: Conferences	2353

Search date: 20 March 2024.

**Table A5:** Nature search strategy.

#	Search	Results
1	(nature or nature aging or nature biomedical engineering or nature nanotechnology or nature neuroscience or nature reviews neurology or nature reviews neuroscience).jn.	131,082
2	“Deep brain stimulation”.ti,ab,kw.	25,484
3	Neuroprosthetic.ti,ab,kw.	913
4	Photostimulation.ti,ab,kw.	1815
5	“Transcranial magnetic stimulation”.ti,ab,kw.	27,828
6	“Transcranial electric stimulation”.ti,ab,kw.	340
7	“Brain–computer interfaces”.ti,ab,kw.	33
8	Brain interface.ti,ab,kw.	462
9	Brain augmentation.ti,ab,kw.	18
10	“Brain computer”.ti,ab,kw.	8439
11	Neurotechnology.ti,ab,kw.	614
12	Cognitive technology.ti,ab,kw.	41
13	or/2–12	64,715
14	1 and 13	221
15	limit 14 to yr = “2023 -Current”	29

Search date: 20 March 2024. Database(s): Embase 1974 to 19 March 2024.

**Table A6:** News Media Scanning Tool search strategy.

#	Source	URL	Results
1	Med-Tech	https://www.med-technews.com	1
2	MedTech Dive	https://www.medtechdive.com	3
3	Medical Device Network	https://www.medicaldevice-network.com	20
4	Medtech Insight	https://medtech.citeline.com	2
5	Medical Device and Diagnostic Industry	https://www.mddionline.com/	0
6	Medical Tech Outlook	https://www.medicaltechoutlook.com/	0
7	MDTechReview	https://www.mdtechreview.com/magazine/	0
8	Today’s Medical Developments	https://www.todaysmedicaldevelopments.com/	0
9	Medgadget	https://www.medgadget.com/	N/A *

Search dates: Lines #1–3 1 January 2023–11 March 2024; line #4–9 1 January 2023–14 March 2024. * URL no longer works. N/A = not applicable.

**Table A7:** GoogleNews Reproducible Search Tool search strategy.

#	Search	Results
1	Neurotechnology	50
2	Cognitive technology	50
3	Neurotechnology mental health	20
4	Neurotechnology healthy ageing	20
5	Neurotechnology disabled adults	13
6	Additional Googles News search (hand-searched)	12

Search dates: 1 January 2023 to 7 March 2024.
